# Does antibiotic awareness campaigns exposure decrease intention to demand antibiotic treatment? Testing a structural model among parents in Western Australia

**DOI:** 10.1371/journal.pone.0285396

**Published:** 2023-05-18

**Authors:** Aaron Lapuz Alejandro, Wei Wei Cheryl Leo, Mieghan Bruce, Kaymart Gimutao

**Affiliations:** 1 Centre for Biosecurity and One Health, Harry Butler Institute, Murdoch University, Murdoch, Australia; 2 School of Nursing and Midwifery, Edith Cowan University, Joondalup, Australia; 3 Fiona Stanley Hospital, Murdoch, Australia; 4 Murdoch Business School, Murdoch University, Murdoch, Australia; 5 School of Veterinary Medicine, Murdoch University, Murdoch, Australia; 6 Developmental Communication, University of the Philippines, Los Baños, Philippines; Public Health Foundation of India, INDIA

## Abstract

Antimicrobial resistance (AMR) is one of the key public health concerns the world is facing today. The effect of antibiotic awareness campaigns (AACs) on consumer behaviour has been documented in the literature with mixed results. Understanding the mechanism for how AACs affect target populations is vital in designing effective and tailored campaigns. Using structural equation modelling our study examined the relationships among people’s exposure to antibiotic awareness campaigns, knowledge of AMR prevention, AMR risk perception, and intention to seek antibiotic treatment. This study also tested the moderating effect of anxiety and societal responsibility on preventing AMR, and on their intention to demand antibiotic treatment mediated by knowledge of AMR prevention and risk-perception. Primary data was generated using an online survey of 250 Western Australian parents. We tested our hypotheses using reliability and validity tests and structural equation modelling. Our results show that exposure to AACs alone may not be enough to change parental intention to demand antibiotic prescription for their children. Parental risk perception of AMR and parental anxiety affect intention to demand antibiotics, and the view that AMR is a social responsibility has a moderating effect on intention to demand antibiotics. These factors could be considered and combine messaging strategies in designing future antibiotic awareness campaigns.

## Introduction

Antimicrobial resistance (AMR) is one of the key public health concerns the world is facing today [1]. By 2050, if no appropriate action is taken it is predicted that ten million people will die every year due to antimicrobial-resistant infections [2]. This grim picture led the World Health Organisation (WHO) to call for national strategies among its member countries to mitigate the spread of AMR. As of 2017, 79 countries had completed their national plan, while 50 others were drafting their plan [3].

One of the key elements of each plan is public education through social marketing and campaigns [4, 5]. One way to achieve public education is through antibiotic awareness campaigns. Promising results have been reported from previous health communication campaigns on AMR. A review of 22 large-scale antibiotic awareness campaigns (AACs) among high-income countries which were associated with reduction in the use of antibiotics and resistance to antibiotics [6]. The latest evaluation of Reducing Antibiotic Resistance in Australia cited an overall reduction of 24.8% in antibiotic use between 2012 and 2017, after a 5-year implementation of a national campaign [7]. These results demonstrate benefits of health communication campaigns in reducing antibiotic prescriptions, however, it is important to note these campaigns targeted healthcare workers as well as the public.

Assessments of the effects of AACs targeting consumers’ behaviour alone have been documented in the literature with mixed results. A study found a modest change in Australians’ beliefs, attitudes, and behaviour in managing upper respiratory tract infections after a national campaign [8]. No improvement in public understanding of the lack of benefit of antibiotics for coughs and colds after conducting a health communication campaign was found in UK study [9]. A study in Poland, reported nearly half of participants claimed their attitude towards antibiotics changed after being exposed to European Antibiotic Awareness Day campaigns from 2009 to 2011 [10]. So, although studies have examined the relationship between AACs and behaviour change, more research is needed to understand the mechanisms of how these campaigns affect individual and population intention and behaviour toward antibiotic use.

Our study used structural equation modelling to examine the relationships among people’s exposure to antibiotic awareness campaigns, their knowledge of how AMR can be prevented, their AMR risk perception, and their intention to seek antibiotic treatment. This study also tested the moderating effect of anxiety and societal responsibility on preventing AMR, and on their intention to demand antibiotic treatment mediated by knowledge of AMR prevention and risk-perception. The study aims to explore effective messages and communication strategies to improve future antibiotic awareness campaigns.

### Literature review and conceptual framework development

Throughout the 20th century, health communication campaigns were considered critical components of intervention efforts to address global health issues [11]. Health communication campaigns can change behaviour both at the individual and societal level [12, 13]. Among individuals, health campaigns can invoke cognitive and emotional responses that directly affect an individual’s decision to adopt a healthy behaviour [12, 14]. At a population level, one’s behaviour change that has become a norm within a social network might also influence another person’s decisions even if they have not been directly exposed to the campaign [12].

#### Campaign exposure and knowledge

Cultivation theory is a key theoretical framework for the study of mass media exposure and its effects on society [15]. Cultivation theory predicts that audience behaviour can possibly be influenced by high exposure to messages on mass media [16]. According to cultivation theory, there is a significant positive association between amount of exposure and message influence on individual’s perceptions of the problem and attitude formation about actions to take [17].

A meta-analysis in 2016 found that health campaigns enhanced the public’s knowledge of health issues, and individuals exposed to the campaigns had a favourable change in their knowledge compared to those who were not exposed to the campaign [18]. Knowledge can influence the comprehension of health issues, future information-seeking behaviours, and disease prevention behaviour in general [19]. Based on these findings, we propose:

H1: AMR campaign exposure increases participant knowledge of prevention of AMR.

#### Campaign exposure and risk perception

Risk perception refers to one’s subjective judgments about the likelihood of negative consequences including injury, illness, disease, and death [20, 21]. Public awareness and perceptions of risk can be influenced by how the media portrays a health issue [21]. Health issues become salient to the public with increased media coverage and in turn, the public will regard the issue as important [22].

Results of a study found an increase in risk perception of acquiring HIV/AIDS upon an individual’s exposure to a health campaign promoting the use of condoms [23]. A multimedia stroke-prevention campaign in Germany resulted in increased numbers of people considering themselves being at risk of developing stroke [24]. In South Africa, a significant recognition of the risks associated with consuming sugary drinks and developing obesity was noted after a campaign on this subject was implemented [25].

Consequently, we propose:

H2: AMR campaign exposure increases participants’ AMR risk perception.

#### Campaign exposure and intention to demand antibiotic treatment

The hierarchy of effects model was developed as part of advertising and marketing theory in the 1960s and was recommended for public health communication in the 1980s [26, 27]. The model posits a causal chain of links between proximal variables (e.g., campaign exposure) and endpoints or distal outcomes (e.g., behavioural change) through a series of intermediate measures (e.g., social norms, attitudes, intentions) [27]. The hierarchy of effects model acknowledges that health campaign success becomes more difficult to achieve as the process moves from initial awareness and knowledge of a health issue to attitudinal and behaviour change [27]. Moreover, the hierarchy of effects model recognises that the proportion of the population that engages in the desired behaviour change will be small even after being exposed to a campaign [28].

One of the common reasons for inappropriate antibiotic prescription for children is parental expectation of an antibiotic treatment [29, 30]. A study conducted in China noted that parental demand for antibiotics contributed to 40% of inappropriate antibiotic use for children [31]. One in every five Italian parents expected an antibiotic prescription for their children prior to consultations [29]. In England, 27% of parents used language that indicated a possible need for and expectation of antibiotic treatment [32]. One in every three Australian parents visits their general practitioners with the intention of getting antibiotics to treat children under 14 years for self-limiting conditions such as sore throats, coughs, and colds [33].

A decrease in parental demand for antibiotic prescriptions has been the target outcome in previous antibiotic awareness campaigns [34, 35]. Results of a systematic review were less than encouraging, finding no significant decrease in parental demands for antibiotic prescription after interventions targeting parents only [34]. For example, no significant difference in antibiotic prescription between the control and intervention group after a poster containing information about judicious antibiotic use was provided in consultation rooms [36]. Another study did not find a significant difference in the number of respiratory tract infection consultations resulting in antibiotic prescription after exposure to a brief videotape message informing parents of indication of antibiotics [37].

More recent interventions, however, show some promising results. An increase in parents’ understanding of the nature of viral illness and the reason for not prescribing antibiotics was found after provision of an information booklet during consultation [35]. Parents reported an increased knowledge of the indications and risks of antibiotics after watching a 90-second animated video on parents’ interest in receiving an antibiotic for their child [38].

Citing these studies, we propose:

H3: Participant knowledge of AMR prevention will decrease participant intention to demand an antibiotic prescription.

#### Campaign exposure and risk perception

Disease risk perceptions are a significant determinant of health behaviour [39]. Studies have demonstrated that most people do not consider themselves at risk of developing AMR infections [40, 41]. Antimicrobial resistance perceived as a serious threat to human health, but ironically, individuals do not perceive themselves to be affected by AMR. In a study among parents, found only a few of parents considered AMR as a potential harmful effect of antibiotic use [41]. Parents also perceived that their family are at a low risk of developing AMR and reported that AMR is a future issue that they are unable to connect with.

The behaviour of individuals significantly influences their health, and how they perceive their risks affects whether they will be motivated to take actions to improve their health [42]. Heightening the risks appraisal in health campaigns influences positive changes among population [43]. Moreover, interventions that were successful in emphasizing risk appraisals of the target population led to changes in subsequent intentions and behaviour [43].

Consequently, we propose:

H4: An increase in participant AMR risk perception will be associated with decrease participant intention to demand antibiotic treatment.

#### Parental anxiety and medical treatment

Parents have reported heightened feelings anxiety when their children are sick [44, 45]. Acute stress such as an illness may impair the evaluation of information critical to decision making and may influence one’s thinking to shift to habit-based pattern rather than a goal-directed conclusion [46]. Another study has associated parental anxiety with decreased trust in their physician, which may leads parents to make autonomous decisions which might be against medical advice [47].

Physicians have cited level of parental anxiety as a factor in their decision whether or not to prescribe antibiotics for children [48, 49]. Parental anxiety during consultations presents a challenge and is often a source of conflict between parents who expect antibiotics and physicians who follow a non-antibiotic prescribing strategy [50]. In addition, due to parental worries, they may present their ‘candidate diagnosis’ during consultation which often implies their expectations for antibiotic treatment [51]. Reduction of anxiety during medical consultations improves increases acceptance of treatments being offered [52]. Alleviating parental anxiety, therefore, may potentially decrease parental demand for antibiotics and increase acceptance of non-antibiotic management.

Based on this literature, we propose:

H5: Participants’ feelings of anxiety if their children were not prescribed with antibiotics will moderate the negative relationships between knowledge of prevention of AMR and AMR risk-perception, and intention to demand antibiotic treatment, such that the negative relationship will be weaker for participants with higher anxiety.

#### Sense of community and AMR prevention

Antibiotics is perceived by the general public to be harmless and a readily available intervention [53]. A tension between individual and collective reasons for engaging in responsible use of antibiotics exists [40]. Antibiotic use poses a social dilemma wherein individuals have to decide whether or not to consider collective interests and AMR prevention when deciding whether or not to use antibiotics [54]. Awareness of a health crisis such as AMR can generate a sense of shared identity and community amongst people, which can lead to increased collective action, acts of solidarity and social accountability [55].

Sense of community is defined as a feeling that members have belongingness, a feeling that members matter to one another, and a shared faith that members’ needs will be met through commitment to be together [56]. Sense of community promotes community development and improves community capacity to solve problems (or likelihood of solving problems) through enhanced internal human resources and promotion of social empowerment [57]. Sense of community promotes protection of citizens during a health crisis [58]. Moreover, sense of community is a vital component of population health prevention strategies that have been positively linked to health-related behaviour changes [59].

Complementing this principle is the view of social accountability which is a widely accepted way to address public health issues such as antimicrobial resistance [60]. Ancillotti et al. [61] posit that there is a societal interest in maintaining antibiotic effectiveness highlighting the role of social responsibility and accountability. Social accountability is defined as an approach toward building accountability that relies on civic engagement, in which it is ordinary citizens and/or civil society organisations that participate directly or indirectly in exacting accountability [62]. Social accountability promotes understanding and a stronger, more trusting relationship between the health system and the community which translates into a higher likelihood of people mobilizing participatory processes and solve problems affecting their community [63].

On the basis of these studies, we propose:

H6: A participant view that AMR is social responsibility will moderate the negative relationship between knowledge of prevention of AMR (6a) and AMR risk perception (6b) and intentions to demand antibiotic treatment such that the negative relationship will be stronger for participants with higher social responsibility.

**Fig 1:**
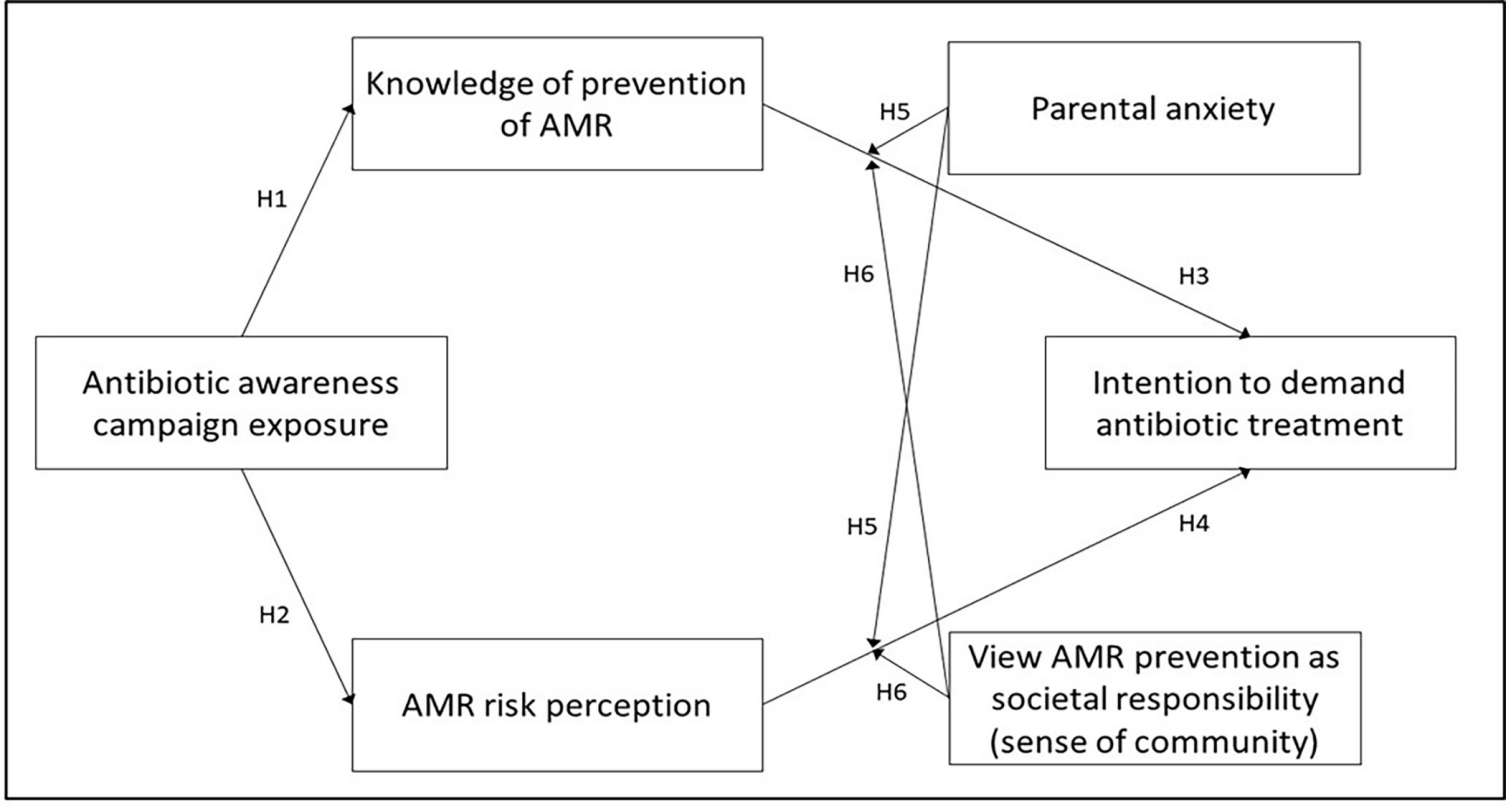
Links between concepts and study hypotheses.

The proposed conceptual framework is illustrated in Fig 1. The present study theorizes that increase in AMR campaign exposure will increase AMR prevention knowledge and participant AMR risk perception and decrease participants intention to demand antibiotic treatment.

## Methods

### Participants

A cross-sectional survey was conducted among 250 parents or caregivers with children under 18 years of age, living in Western Australia. The survey was administered as part of a larger study that explores the effect of the COVID-19 pandemic on parents’ preventative health behaviour for their children.

### Material

The survey was divided into two parts. The first part collected demographic information about the participants. The second part measured the variables in the research model using 5-point Likert scale. The questionnaire was delivered in English.

The initial questionnaire was randomly given to five individuals: three parents and two nurses. The questionnaire was revised to incorporate their input. Ten completed pilot surveys were assessed for validity. No further revisions to the questionnaire were made after the review of the pilot surveys.

Participants responded using 1–5 Likert scales (strongly agree to strongly disagree). Constructs were measured through items adapted from previous literature to meet the requirements of this study. To assess the amount of media exposure to antibiotic awareness campaigns, participants were asked to indicate if they had seen antibiotic awareness campaigns in mainstream media, and in community and general practices they attend. Six items to measure the knowledge of participants regarding proper antibiotic use and prevention of AMR were adopted based on Australian Government recommendations [64]. Four items of AMR risk perception were modelled using the Health Belief Model [65] and previous studies by [66]. For moderating factors, participants were asked to rate their feelings of anxiety if their children did not receive an antibiotic prescription [48]; and their view on whether AMR prevention is a societal responsibility [40]. All survey items are presented in Supplementary Material A. Terms, definitions, and abbreviations for the study variables are summarised in Table 1.

**Table 1:** Definition of variables.

Variable	Abbreviation	Definition
AMR Campaign exposure	AE	The amount of the parents’ exposure to AMR campaigns in mass media, community and medical practices they attend.
AMR Knowledge	AK	The knowledge of parents about indication of antibiotics and prevention of AMR.
AMR Risk	AR	The perceived risks of AMR.
Intention to demand antibiotic treatment	IA	The intention of parents to demand antibiotic treatment for influenza-like-illness
Feelings of anxiety	FA	The feeling of anxiety of parents if there is no prescription of antibiotics.
Sense of community	SR	The belief of parents that preventing AMR is a social responsibility.

### Procedure

Data were collected from October 21 to November 27, 2020, by a private research company, Pureprofile (https://www.pureprofile.com/). Participants were recruited through the Pureprofile panel. Invitations to participate were sent online and recruitment continued until the required 250 participants was reached. Responses were voluntary and anonymous. Participants had no direct contact with the research team.

### Ethics approval

This study was approved by Murdoch University’s Human Research Ethics Committee, approval number 2020/118. A written informed consent was gained from participants prior to commencing the online survey.

### Statistical analysis

Descriptive statistics was conducted using SPSS 23.0 (IBM, Armonk, NY). To test our hypothesis, we used SPSS AMOS 27. First, we tested the reliability of the items used for measuring model constructs: AMR campaign exposure, AMR knowledge, and AMR risk-perception, using Cronbach’s alpha value. For this study, we have accepted Cronbach’s alpha value of greater than 0.6 following the general rule that α of 0.6–0.7 indicates an acceptable level of reliability [67, 68].

Secondly, we ran confirmatory factor analysis to measure the strength of the influence or the correlation of the scores of items with the scores of the constructs and was determined based on the magnitude of the factor loading of each item. We adopted Hair’s factor-loading threshold of less than 0.32 (poor), 0.33–0.45 (fair), 0.46–0.55 (good), 0.56–0.69 (very good), ≥ 0.70 (excellent) [69].

Following that, we ran structural equation modelling (SEM) to test the fit of our proposed model and test our hypotheses within a structural model. For this study, we have adopted the goodness-of-fit criteria proposed by [70] using chi-square (model acceptable if P< 0.05) and other fit indices including Comparative Fit Index (model acceptable if CFI > 0.90), Tucker–Lewis Index (model acceptable if TLI > 0.90), and Root Mean Square Error of Approximation (model acceptable if RMSEA is < 0.08).

A multi-group analysis was conducted to test for the moderating hypotheses (H5 and H6). Participant responses to feelings of anxiety and sense of community were dichotomised using the median split approach [71]. Significant relationships among the variables were examined using bootstrapping procedures, which resampled distribution by 5,000 with 95% confidence intervals.

## Results

### Description of the participants demographic description of participant cohort

**Fig 2:**
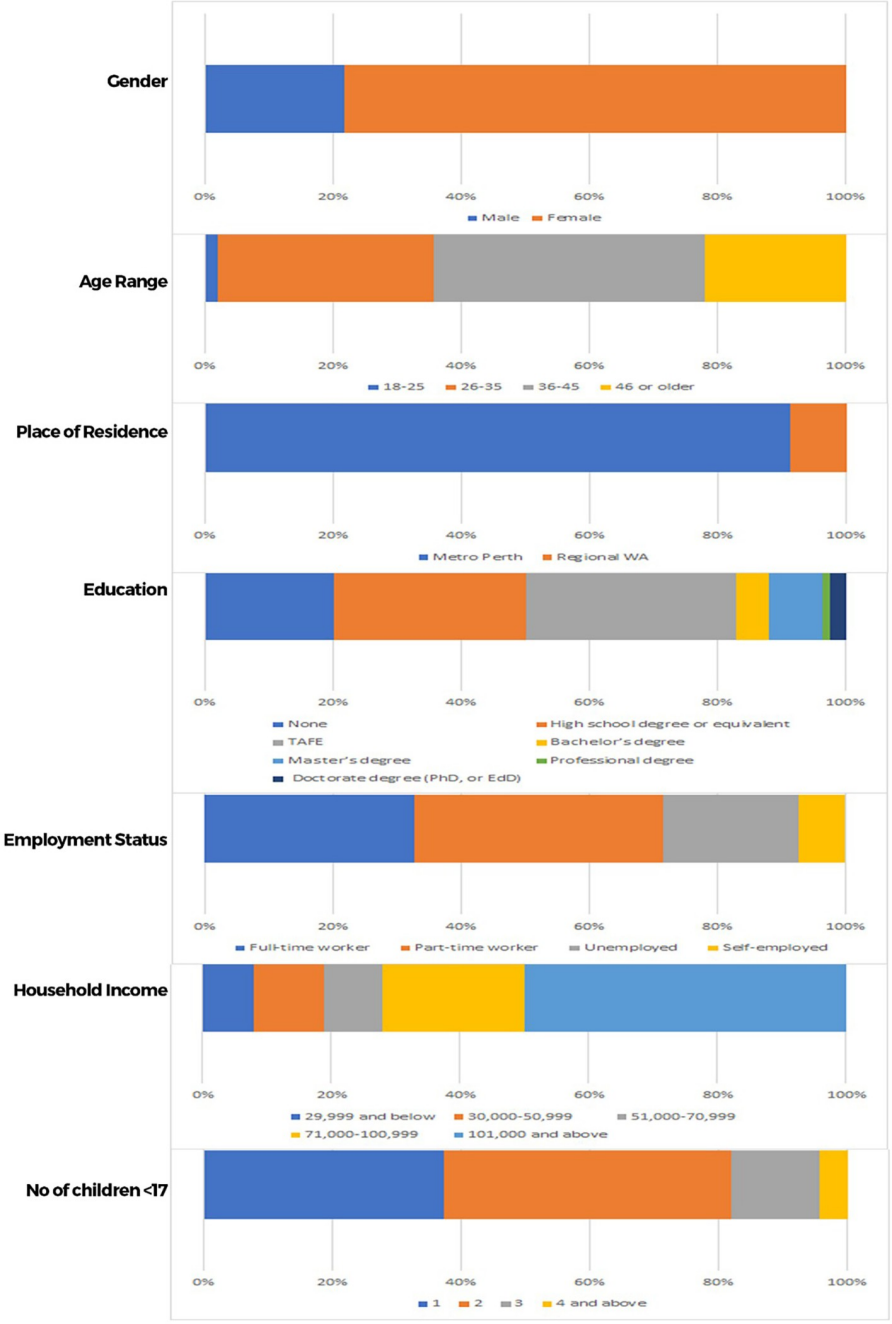
Demographic characteristics of participants.

Uneven gender distribution existed in the sample, with a larger cohort of female participants (n = 196, 78%) compared to male (n = 56, 22%). Most of the participants were in the 36–45 years age range (n = 106, 42.4%), followed by 26–35 years age range (n = 84, 33.6%); 46 and older (n = 55, 22%); and 18–25 range (n = 5, 2%). The participants in this study were mostly from the Perth metropolitan area (n = 228, 91%), with a small number from regional Western Australia (n = 22, 9%). Educational background varied: 30 (12%) completed Master’s degree; 13 (5%) have a bachelor’s degree; 82 (32%) completed technical or further education certificate; 75 (30%) completed a high school diploma; and 50 (20%) do not have any education certificate. Most of the participants were employed (n = 197, 79%); of those 39% (n = 97) were working part time; 32% (82) were working full time, and 7% (18) were self-employed. Half (n = 125) of the participants had yearly household income of $101,000 and above; 22% (n = 55) earn between $71,000-$100,999; 10.8% (n = 27) earn between $30,000-$50,999; 9.2% (n = 23) earn between 51,000-$70,999 and 8% (n = 20) earn $29,999 and below. At 44.8%, almost half of the participants have two children (n = 112), 37.2% have one child (n = 93), 13.6% have three children (34) and 4.4% have four children and above (11). Participant demographic characteristics are summarised in Fig 2.

### Descriptive analysis of variables

The descriptive statistical results of items and constructs were shown in Table 2.

**Table 2:** Results of variable items.

Constructs	Items	Mean	Std deviation
**Antibiotic awareness campaigns exposure**	**AE1**	2.78	1.11
	**AE2**	2.50	0.99
	**AE3**	2.57	1.13
**Knowledge of prevention of AMR**	**AK1**	3.33	0.96
	**AK2**	3.55	0.90
	**AK3**	3.85	0.95
	**AK4**	3.58	1.00
	**AK5**	3.39	1.03
	**AK6**	3.60	1.06
**AMR risk-perception**	**AR1**	3.35	0.84
	**AR2**	3.73	0.86
	**AR3**	3.30	0.84
	**AR4**	3.58	0.84
**Intention to demand antibiotic treatment**	**BI1**	2.47	1.09
**Feelings of anxiety**	**FA1**	2.50	1.07
**Sense of community**	**SR1**	3.67	0.84

### Reliability of the constructs

The three main constructs (campaign exposure, AMR knowledge, and AMR risk-perception) used in this study had Cronbach’s alpha value of more than 0.6 which is acceptable according to [68]. The following are the Cronbach’s alpha value for each construct: α = 0.87; α = 0.78, and α = 0.646. In the final model, three statements from AMR knowledge (AK1, AK2 and AK3) were eliminated based on the item-to-total statistics. We then retested the reliability of the AMR knowledge using the four remaining statements which resulted in a Cronbach’s alpha of 0.76.

### Validity of the items and model fit

A confirmatory factor analysis was performed to evaluate validity of the items in each construct. After running the initial model with all of the items of each construct, three items (AK1, AK2 and AK3) from AMR knowledge construct yielded factor loadings of less than 0.6 and were removed from the final model. The remaining factor loading were all above the very good cut-off (0.56–0.69).

Overall goodness-of-fit of the SEM model was evaluated to determine its suitability for analysing the effect of antibiotic awareness campaign exposure on intention of parents to demand antibiotic treatment for their children. Overall, our model met the goodness-of-fit criteria [70], as summarised in Table 3.

**Table 3:** Summary of goodness-of-fit.

Goodness-of-fit Criteria	Measurement Standard (70)	Fitted value
Chi-square	> 0.05	>0.01
Comparative Fit Index	> 0.90	0.96
Tucker–Lewis Index	> 0.90	0.95
Root Mean Square Error of Approximation	< 0.08	0.06

### Structural equation modelling path analysis

H1 posited a positive relationship between antibiotic awareness campaign exposure and participant knowledge of prevention of AMR. We found no significant positive association between campaign exposure and participant knowledge of AMR prevention (β 0.142, p 0.065). This means that participants’ exposure to antibiotic awareness campaigns did not improve their knowledge of antimicrobial resistance prevention.

H2 posited a positive relationship between AMR campaign exposure and AMR risk perception. We found campaign exposure had positive and significant association with participant risk perception of AMR (β 0.223, p 0.0004). This means that participants’ exposure to antibiotic awareness campaigns improve their risk perception of antimicrobial resistance.

H3 posited that knowledge of AMR prevention will affect participants’ intention to demand antibiotic treatment for their children. We found a significant positive relationship between participant knowledge of prevention of AMR and intention to demand antibiotic treatment for their children (β .243, p < .001). This means that even if participants are knowledgeable in preventing AMR, it will not stop their intention to demand antibiotic treatment for their children.

H4 posited that higher AMR risk perception will decrease participants’ intention to demand antibiotic treatment for their children. We found a significant negative relationship between AMR risk perception and intention to demand antibiotic treatment (β -.411, p < .001). This means that participants with higher perceived risk of AMR have a lower intention to demand antibiotics for their children.

### Multi-group analysis and moderating effects in the SEM

H5 posited that participant feelings of anxiety will have a moderating effect on participant intention to demand antibiotic treatment. Participants’ anxiety did not have a significant moderating effect on intention to demand antibiotic treatment, regardless of their knowledge of AMR prevention. However, participants’ AMR risk perception was moderated by their feeling of anxiety and affected their intention to demand antibiotic treatment (p < .05). Participants who had a low feelings of anxiety when their child was not prescribed antibiotics had negative relationship with their intention to demand for antibiotic treatment (β -.450, p < .001). In contrast, participants with high feelings of anxiety when their child is not prescribed with antibiotics had weaker relationship with their intention to demand for antibiotic treatment (β -.173, p.229). This may imply that participants with high AMR risk perception and low feelings of anxiety without antibiotic prescription are least likely to demand antibiotic prescription compared to those who have high feelings of anxiety.

H6 posited that a participant view that AMR is a societal responsibility will have a moderating effect on the relationship between participants’ knowledge and intention to demand antibiotic treatment for their child. Results demonstrated that there is no moderating effect for this relationship. However, AMR risk perception was moderated by a view that AMR is a social responsibility, thus moderates the relationships between risk perception and intention to demand for antibiotic treatment (p < .05). Results also implied that participants who had high belief that AMR is a societal responsibility had a stronger negative relationship between AMR risk perception and their intention to demand antibiotic treatment (β -0.407, p < .001). In contrast, there was a weaker relationship between risk perception and intention to demand antibiotic treatment (β 0.041, p 0.736) for participants with lesser belief that AMR is a social responsibility. This implies that participants who believed that AMR is a societal responsibility are least likely to demand antibiotics when they perceive high AMR risks, as compared to those who did not believe that AMR prevention is a social responsibility.

## Discussion

This study used structural equation modelling to examine the effects of antibiotic awareness campaign exposure on parental knowledge of prevention of AMR, AMR risk perception, and intention to demand antibiotic treatment for their children. Additionally, using multi-group analysis, the moderating effects of parental anxiety and sense of community on parents’ intention to demand antibiotic treatment for their children were examined.

Results show that antibiotic awareness campaign exposure did not improve parent knowledge of prevention of AMR. This finding contradicts our first hypothesis and previous studies linking campaign exposure and increased knowledge of prevention of AMR [10, 72, 73]. Our findings have similarities with a systematic review which found no improvement in antibiotic-related knowledge among a population with the use of mass-media campaigns that targeted both the public and clinicians [74].

Knowledge of prevention of AMR was also found not to decrease parents’ intention to demand antibiotic treatment for their children. This indicates that provision of information to improve knowledge may not lead to behavioural change. Solely increasing the public’s knowledge about antibiotic use may actually be counterproductive with respect to self-medication [75]. This observation has been seen in China wherein more educated Chinese self-medicated with left-over antibiotics instead of going to their general practitioners when they had respiratory tract infections [76]. Another study found that participants who attended an antibiotic awareness workshop took twice the amount of antibiotics after compared to before the campaign [77]. Knowledge gain from campaign exposure may therefore have unintended consequences [78]. Inaccurate and poorly designed health communication campaigns might even do more harm than good to the target audience [79].

One major gap in these AMR campaigns is the lack of application of behavioural and social sciences, which have been applied and have greatly contributed to other public health areas [80, 81]. Previously it has been argued that traditional AMR campaigns, that are grounded in information-intensive health education approaches, do not lead to sustainable behaviour change [82]. Moreover, some educational campaigns assume the population lacks knowledge and that providing them with knowledge will alter their behaviour [83]. Often this results in one-size-fits-all approaches to campaigns, with objectives that are not relatable to individual needs, resulting in campaign failure [84].

One way of addressing this gap is to understand consumers’ behaviour which a campaign is targeting through customer orientation [85, 86]. Customer orientation is central to the health communication planning process, often through formative research, pretesting, and pilot testing that is used to gain a deeper understanding of a target audience’s needs, values, behaviours, and everyday lives [85]. A lack of customer orientation has been seen in previous antibiotic awareness campaigns among parents [35, 41]. In Netherlands, an information booklet regarding antibiotic use did not change parent attitudes as they already knew the information contained in the booklet [35]. The “Keep Antibiotics Working” campaign in the UK did not address misconceptions of parents regarding antibiotics use, specifically parents who are “low users” of antibiotics. British parents sought better public campaign strategies on AMR, utilising messages that are relevant for them and their families, and that match their daily lives [41]. This indicates the need for customer orientation, which generates interest in changing behaviour among the target audience, and motivates them to voluntarily change behaviour and sustain the change [87].

One interesting result of our study is that increased AMR risk perception decreased parents’ intention to demand antibiotic treatment. A national survey indicates that more Australians believe antibiotic resistance is affecting them and their family compared to previous years [88]. On the other hand, our findings are in contrast with studies conducted in the UK and the USA [35, 53]. In these studies, participants’ proclivity towards antibiotics did not change even if they were aware of the risks of AMR. Our findings suggest that communicating the risks of AMR could deter parents from demanding antibiotics for their children.

Interventions that change risk perception subsequently change health behaviours [39]. One study found that parents’ perception of child risk for future health problems was a strong predictor of parent readiness to change a behaviour [89]. Moreover, perceived risk is a key element in individuals adopting preventative behaviour and seeking health information [90, 91]. Unfortunately, parents may have an inaccurate risk assessment of AMR. According to a study in the USA by [92], for example, the majority of parents were not concerned about antibiotic resistance. Only few parents in the UK considered antibiotic resistance as a possible health risk, and considered their families less likely to develop AMR due to low usage of antibiotics [41]. In Australia, parents viewed AMR as a problem but perceived that it would not impact them individually [93]. AMR has been viewed as a distant and future problem resulting in low-risk perception among individuals [40, 53, 93]. In psychology, this phenomenon is known as “psychological distance”.

Psychological distance is defined as the subjective experience that something is closer or far away from the self, and present [94]. Psychological distance falsely lowers an individual’s perception of risk severity and susceptibility. Thus, individuals might not alter their behaviour even though they could make a difference [95]. Bridging this psychological distance presents a unique challenge to antibiotic awareness campaigns. Previous AMR narratives had depicted AMR risks with a distant focus such as “doomsday,” “post-antibiotic apocalypse,” and “future catastrophe’ [96–98]. These narratives could further increase psychological distance, making AMR communication counterproductive, as individuals have a higher propensity to perform positive behaviours when an issue is perceived as more proximal and concrete to them [99]. Communicating current risks and present impact of AMR might be more effective in antibiotic awareness campaigns.

Our findings also showed the moderating role of parental anxiety in intention to demand antibiotics for their children. In our study, parents who had higher AMR risk perception and lower feelings of anxiety had lower intention to demand antibiotics as compared with parents with higher feelings of anxiety. This finding highlights the need to address parental anxiety during consultations. A previous study has shown that general practitioners may prescribe antibiotics in order to reassure anxious parents and to relieve their own anxiety [100]. One study suggested that doctors’ use of “running commentary” is useful in modifying parent expectation of antibiotics during consultations. Running commentary allows sharing of information between parents and physicians, in a reassuring manner, which can potentially decrease parental anxiety [50], and therefore potentially reduce the intention of parents to demand antibiotics. In terms of AMR campaigns, conveying empowering messages to parents, including information that upper respiratory tract infections symptoms are self-limiting and can easily be self-managed, can decrease intention to demand antibiotics [101]. Empowering parents has also been linked to decreased parental anxiety and enhanced parental confidence in managing their sick children [102].

Lastly, our study found that a parent holding the view that AMR prevention is a societal responsibility had a moderating effect on their intention to demand antibiotics. Our finding support results of a study indicating that individuals with an altruistic view of society engage in judicious use of antibiotics [40]. In our study, parents who had a higher sense of community and social responsibility had a lower intention to demand antibiotics. This finding implies that antibiotic awareness campaigns will be more effective if they promote the attitude/idea that AMR prevention is everyone’s responsibility and affects us universally [103]. Previous communication about the consequences of AMR has primarily focused on the health consequences of vulnerable groups rather than society as a whole [104]. Moreover, AMR communication has been framed as a human health issue, with messages that target individual clinical encounters and antibiotic misuse rather than wider societal action [105]. Health messages that emphasised societal benefits, rather than focusing solely on the individual, persuaded more individuals to engage in preventative behaviour and also motivated others to do so [106]. There is significant support therefore, in our research and others’, for the proposal that highlighting AMR prevention as a pro-social behaviour could reduce parent intention to demand antibiotics.

### Limitations and future research

Future research could consider an actual campaign on AMR specifically designed considering the model and constructs in this study. A single, tailored campaign can serve as the basis of a study to investigate respondents’ knowledge [107], attitude [93], and practice/s of antibiotic use. It would be it would be more accurate to measure the effects of a campaign on knowledge, attitude, and behaviour of participants, if a control group of participants were exposed to a tailored campaign, as opposed to trying to measure the effect of various campaigns to gauge the knowledge gained by participants from a tailored campaign. Our study depended on campaigns participants were previously exposed to. These campaigns were diverse in the way they framed campaign messages, and the mode (mass or interpersonal) and medium (e.g., radio, television, posters) of communication used. It can be assumed that participants from our study, therefore, have had different exposure to messaging targeted at changing their level of knowledge, perception of risk, and current practices with respect to antibiotic use and AMR. Future studies that use a single campaign specifically designed/developed for the research is thus highly recommended. Future studies assessing the most effective mode and medium of campaigns (e.g., face to face, use of audio–visual media) could help improve campaign effectiveness. Furthermore, our instrument lacked specification of the media where participants exposed to. Future studies may consider determining the effect of specific media types such as television, radio, newspaper, and billboard that the respondents were exposed to. This may generate richer data and further may further explore the role of Cultivation theory in antibiotic awareness campaigns.

## Conclusion

This structural model highlights that AMR campaign exposure alone may not be enough to change parental intention to demand antibiotics. Several moderating factors, identified in this research, affect parental expectation to demand antibiotics for their children. These complex interactions could be further explored and new knowledge, from current and future studies, utilised to improve the effectiveness of AMR communication in reducing demand for antibiotics and reduce AMR overall.

This research highlighted that knowledge does not always translate to behaviour change. Exploring the barriers that prevent adaptation of responsible use of antibiotics may contribute to better messaging and communication strategies. Understanding the target audience may contribute to a more tailored campaign messages which may results to more successful campaigns. Thus, antibiotic awareness campaigns could utilise behavioural theory such as social marketing may be more effective than traditional campaigns focusing on information provision and one-size fits all approach.

The model suggested that people’s awareness of an issue negatively affecting society may influence them to adopt healthy behaviours. Parental anxiety regarding their child’s illness be acknowledged and addressed during consultations and communication that empowers parents may potentially deter intention of parents to demand antibiotics.
